# Defining the Terminology for Acute Ear Discharge in Children: Systematic Review and Expert Consensus Using the Nominal Group Technique

**DOI:** 10.1111/coa.70021

**Published:** 2025-08-06

**Authors:** Elliot Heward, James Dempsey, Amy McGovarin, John Molloy, Mark Wilbourn, Eason Sivayoham, Merijam Kikic, Jaya R. Nichani, Iain A. Bruce

**Affiliations:** ^1^ Division of Infection, Immunity and Respiratory Medicine, School of Biological Sciences, Faculty of Biology, Medicine and Health University of Manchester Manchester UK; ^2^ Royal Manchester Children's Hospital, Manchester University Hospitals NHS Foundation Trust Manchester UK; ^3^ Windsor Medical Centre St Helier Jersey; ^4^ Paediatric Audiology Department Fairfield Hospital, Northern Care Alliance Bury UK

## Abstract

**Background:**

Acute otitis media (AOM) is one of the most frequent paediatric infections. In approximately 15% of children with AOM, the tympanic membrane perforates, leading to ear discharge. This subset of children is usually more unwell and may need different treatment than those without a perforation. Therefore, terminology is required to differentiate this population from AOM without perforation. Using a consensus exercise, we aimed to standardise disease terminology for this patient group.

**Methods:**

A systematic review was performed using OVID Embase to identify the terminology used within the current published literature. The RAND version of the Nominal Group Technique was then used to gain consensus using an expert panel.

**Results:**

The systematic review identified 2012 abstracts which were reviewed, of which 29 manuscripts were included. A total of nine different definitions were identified within the literature. The expert panel concluded that the terminology ‘acute otitis media with discharge (AOMd)’ should be used when diagnosing a child with an acutely discharging ear of up to 6 weeks duration. Recurrent disease should be diagnosed when four or greater episodes occur per year. Within this disease context, the panel determined the optimal research question was to identify the best management option.

**Conclusion:**

This consensus process has proposed the terminology that should be applied for children with acute ear discharge secondary to AOM. The use of standardised terminology is essential to improve patient care and ensure homogeneity across future research.

**Summary**

- Standardisation of disease terminology is important for clinical and research settings.
- ‘Acute Otitis Media with Discharge (AOMd)’ should be the terminology used to describe acute ear discharge in children caused by a middle ear infection
- AOMd can be used when diagnosing a child with an acutely discharging ear of up to 6 weeks duration.
- Recurrent disease should be diagnosed when four or greater episodes occur per year.
- Future research should determine the best treatment for AOMd

## 1 Introduction

Ear discharge in children is most frequently secondary to acute otitis media (AOM). Ischemia of the tympanic membrane caused by a middle ear infection leads to perforation and otorrhoea [1]. The incidence of childhood ear discharge presenting to primary care is 2.42 children per 1000 patient‐years costing the NHS £2 million per year [2].

There is a physical, social, and psychological impact on children with discharging ears and their family [3]. Some children with ear discharge can have a more severe illness compared to those without, which is contrary to the traditional belief that drum perforation leads to symptom relief [4]. These findings are limited to a single publication from Smith et al. who showed children with a perforation had higher temperatures and pulse rates (Adj OR 1.8 and 1.09) compared to those children without a perforation. Serious complications can occur, with intra‐or extra‐cranial and intratemporal spread of infection. Children suffer with a resultant hearing loss during a critical time, within the first 3 years of life, for language development [5]. The treatment options are different for those with and without a perforation. Those with a perforation can be treated with topical or oral antibiotics and water precaution advice. Otolaryngology doctors based in the UK appear to prefer topical antibiotics compared to oral antibiotics which are favoured in primary care [3].

In the authors' experience, the terminology used to describe children with acute ear discharge is inconsistent. Frequently, the umbrella term of AOM is used for children with or without ear discharge. However, there are clear differences in terms of disease impact and treatment options when comparing AOM with discharge and without discharge. The terminology should therefore allow differentiation between these two subsets of AOM to ensure appropriate patient management and improve research standardisation. The primary aim of this research was to use a consensus exercise to determine the appropriate terminology using a group of experts in the UK. The secondary aim was to identify research priorities listed in the literature and according to the expert panel.

## 2 Methods

**FIGURE 1:**
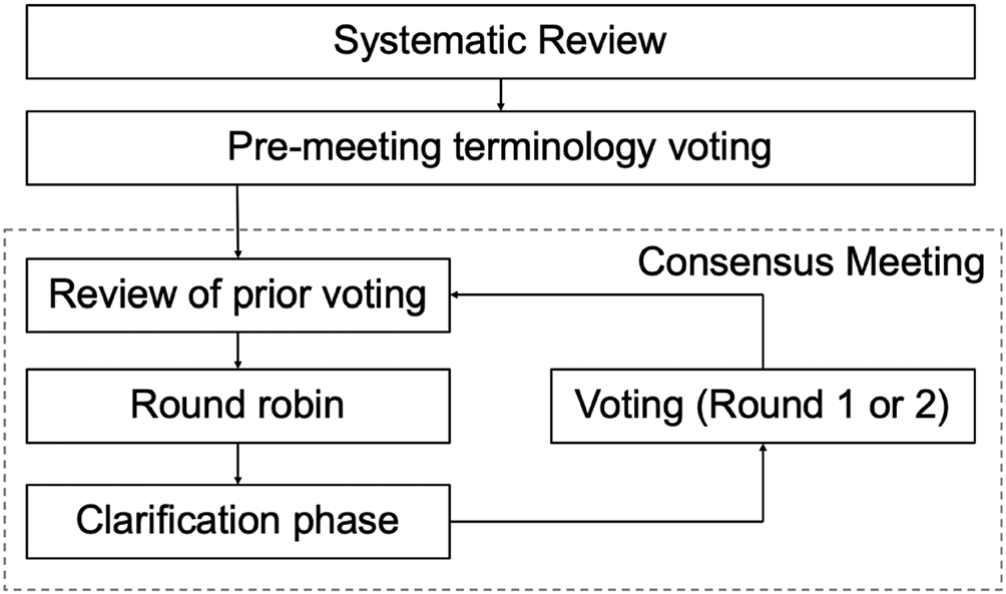
Consensus exercise.

This study is published in accordance with the ACCORD reporting guidelines for consensus studies [6]. The RAND modification of the Nominal Group Technique was identified to be the most appropriate consensus method because of the niche subject area [7]. The process outlined by McMillan et al. was followed (Figure 1) [8]. A maximum of seven panellists is suggested using this technique [9].

The consensus research questions were: (1) ‘What terminology should we use to describe acute ear discharge secondary to a middle ear infection?’ (2) ‘What time frame differentiates acute from chronic discharge?’ (3) ‘How many episodes over what time period represents recurrent infections?’. Panellists were also asked to list the key priorities for future research for this condition, separate to the consensus meeting.

### 2.1 Systematic Review

A systematic review was registered with PROSPERO (CRD420250593067). A search on OVID Embase was performed on the 7th of December 2024. The search strategy can be found on PROSPERO. OVID Embase was used because it is a comprehensive database and covers Medline records. Searching of references within included manuscripts was also performed. All study types and all manuscript languages on this topic were included from 1st January 1946 to 6th December 2024. Titles and abstracts were reviewed by E.H. and J.D. Disagreements were agreed upon. Full text review was performed for included papers. Extraction of data was collated on Microsoft Excel. Disease terminology, definition of acute versus chronic, what determines recurrent disease, and future research priorities were collected. Similar definitions were grouped. Study quality assessment was performed using the Quality Assessment for Diverse Studies (QuADS) tool by E.H. and J.D. [10]. This tool was used because of the varied methodological design of the included studies. Each criterion is assessed on a scale of 0–3 which relates to the clarity of information included in the study. Broadly, zero equates to no information and three to a clear explanation.

Meta‐analysis was not possible due to the categorical nature of the data extracted from the studies.

### 2.2 Selection of the Expert Panel

E.H. and I.A.B. were responsible for selecting the panellists. Panellists were selected from medical specialities who frequently manage children with acute ear discharge. Medical professionals who had previously published literature in this field or those with a specialist interest were invited to join the panel (Table 1). A parent with lived experience of caring for their child with acute ear discharge was also invited to join the panel.

**TABLE 1:** Expert panel members.

Panellist	Role	Gender
EH	Otolaryngology registrar and doctoral researcher in subject area	Male
JRN	Paediatric otolaryngology consultant	Female
MW	Consultant general practitioner with interest in otolaryngology	Male
MK	Consultant paediatrician with interest in paediatric audiology	Female
JM	Consultant paediatric immunologist	Male
AM	Parent with lived experience	Female

### 2.3 Assessing Consensus

One nominal group was used with seven expert members and one facilitator; the session was recorded. Panellists were sent the research questions and information about the consensus process in advance of the consensus meeting. They were asked to select the most appropriate terminology from the list of terms identified from the literature review or generate a new term. Results were collected on Google Forms.

A consensus meeting was conducted virtually (Microsoft Teams) on Wednesday 29th of January 2025 to allow participants to attend from all areas of the UK. The results of the pre‐meeting questions were reviewed. Participants took it in turns during the round robin phase to explain their thoughts relating to each question. The clarification phase involved free discussion.

Participants voted anonymously on the three research questions on Google Forms. Terminology was rated using a nine‐point scale: 1 = extremely inappropriate, 5 = uncertain, and 9 = extremely appropriate. The consensus definition was based on the work by Murphy et al. [7]. The definition of consensus was five of six participants voting in the same range (e.g., 1–3 or 4–6 or 7–9). Disagreement was defined as two or more participants voting in the contrary range to the other participants (e.g., 1–3 and 7–9). Any other outcomes were defined as equivocal.

Following voting, the results were reviewed by the panellists. Questions meeting consensus were removed. Questions not meeting consensus entered into a second round of round robin and clarification before voting. A limit of three rounds was given to achieve consensus.

### 2.4 Research Priorities

Research priorities were extracted from studies included in the systematic review. The expert panel members were asked to outline research priorities using a free text response. These responses were then grouped.

## 3 Results

### 3.1 Systematic Review

**FIGURE 2:**
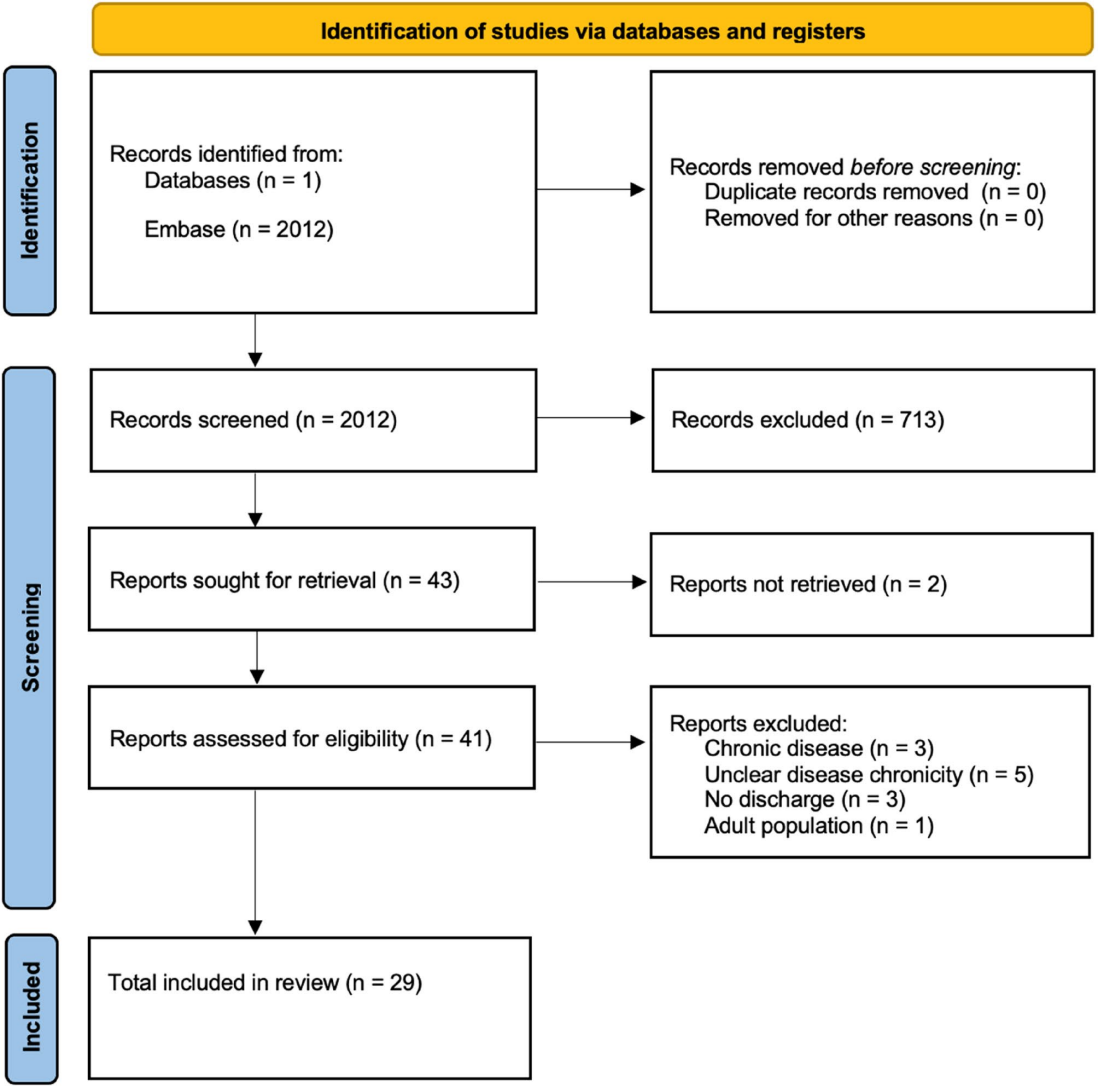
Prisma flow diagram.

The search identified 2012 papers, of which 29 were included (Figure 2). Study quality assessment is outlined in Table 2. No studies were excluded following quality assessment. Nine different terms were identified within the literature describing acute ear discharge (Table 3). Eight papers described a time scale which defined acute ear discharge (≤ 3 days *n* = 2, ≤ 7 days *n* = 3, ≤ 14 days *n* = 1 and ≤ 6 weeks *n* = 2). A definition of recurrent disease was included in three papers. Identifying the best antibiotic management was the most frequent (*n* = 5) research priority listed, followed by monitoring of antimicrobial profile and resistance (*n* = 4).

**TABLE 2:** Study quality assessment (Scale: 0 = no information—3 = clearly outlined).

Author	Publication year	Methodology	Underpinning of research	Research aims	Research setting	Study design	Sampling	Rationale for data collection	Data collection	Data collection procedure	Recruitment	Justification of analytic methods	Analytic methods	Research stakeholder inclusion	Strengths and limitations
Heward [3]	2024	Qualitative	3	3	1	3	2	3	3	3	2	2	3	3	3
Hullegie [11]	2024	RCT	3	3	3	3	2	2	2	3	2	3	3	3	3
Cohen [12]	2023	Cross Sectional	3	3	1	3	1	3	3	3	1	3	3	0	1
Levy [13]	2023	Case Control	3	3	1	3	1	2	3	3	1	3	3	0	3
Assad [14]	2023	Cross Sectional	3	3	2	3	2	3	3	3	1	3	3	0	3
Hullegie [15]	2022	Review	3	3	—	—	—	—	—	—	—	—	—	—	—
Hullegie [11]	2021	Protocol	3	3	2	3	3	2	3	3	2	3	3	3	3
Hay [16]	2021	RCT	3	3	3	3	3	3	3	3	3	3	3	3	3
Xie [17]	2021	Cross Sectional	3	3	2	2	1	1	1	1	2	2	3	0	1
Hullegie [18]	2021	Systematic Review	3	3	—	3	3	2	3	3	—	3	3	0	3
Curtis [19]	2020	Protocol	3	3	3	3	3	2	3	3	3	3	3	3	1
Filipe [20]	2020	Cross Sectional	3	3	3	3	3	3	2	3	3	2	3	0	3
Levy [21]	2019	Cohort	3	3	3	3	2	3	3	3	3	3	3	0	3
Principi [22]	2016	Review	3	3	—	—	—	—	—	—	—	—	—	—	—
Sonsuwan [23]	2016	Cross Sectional	3	3	2	3	3	2	2	2	3	3	3	0	3
Venekamp [24]	2016	Review	3	3	—	—	—	—	—	—	—	—	—	—	—
Yunfang [25]	2015	Cross Sectional	3	3	3	3	3	2	3	3	3	3	3	0	3
Rodrigues [26]	2013	Cross Sectional	3	2	3	2	3	2	2	2	2	2	3	0	1
Chen [27]	2013	Retrospective Observational	3	3	2	3	2	3	2	2	3	2	3	0	3
Marchisio [28]	2013	Retrospective Observational	3	3	3	3	2	2	3	3	2	3	3	0	1
Smith‐Vaughn [29]	2013	Cross Sectional	3	3	3	3	2	3	3	3	2	3	3	0	3
Grevers [30]	2012	Cross Sectional	3	3	3	3	3	2	1	1	3	0	0	0	3
Marsh [31]	2012	Cross Sectional	3	3	3	3	2	2	1	1	2	0	0	0	3
Neumark [32]	2011	Cohort	3	3	3	3	3	3	3	3	3	3	3	0	2
Stamboulidis [33]	2011	Case Control	3	3	3	3	3	3	2	2	3	3	3	0	3
Smith [4]	2010	Cohort	3	3	3	3	2	3	3	3	2	3	3	0	3
Leibovitz [34]	2009	Retrospective Observational	3	3	3	3	1	2	1	1	2	3	3	0	1
Al‐Shawwa [35]	2005	Retrospective Observational	3	3	2	2	2	3	2	2	2	0	0	0	1
Berger [36]	1989	Cohort	3	3	2	3	1	2	2	2	1	0	0	0	1

Abbreviation: RCT, Randomised controlled trial.

**TABLE 3:** Terminology used in publications investigating acute paediatric ear discharge.

Author	Publication year	Terminology	Timescale for acute ear discharge	Definition of recurrent disease	Research priorities
Heward [3]	2024	Acute otitis media with discharge (AOMd)	—	—	Best antibiotic treatment
Hullegie [11]	2024	Acute otitis media and discharge (AOMd)	—	—	—
Cohen [12]	2023	Acute otitis media with spontaneous perforation of the tympanic membrane (AOM with SPTM)	—	—	—
Levy [13]	2023	Spontaneous perforation of the tympanic membrane (SPTM)	—	—	—
Assad [14]	2023	Acute otitis media with spontaneous perforation of the tympanic membrane (AOM with SPTM)	—	—	Monitoring of antimicrobial profile and resistance
Hullegie [15]	2022	AOM presenting with discharge (AOMd)	—	—	—
Hullegie [11]	2021	Acute otitis media with discharge (AOMd)	—	—	—
Hay [16]	2021	Acute otitis media with discharge (AOMd)	≤ 7 days	—	Best antibiotic treatment
Xie [17]	2021	Acute suppurative otitis media (ASOM)	—	—	Causative factors
Hullegie [18]	2021	Acute otitis media with spontaneous perforation of the tympanic membrane (AOM with SPTM)	—	—	Monitoring of antimicrobial profile and resistance
Curtis [19]	2020	Acute otitis media with discharge (AOMd)	≤ 7 days	—	—
Filipe [20]	2020	Acute otitis media (AOM)	< 14 days	—	—
Levy [21]	2019	Spontaneous perforation of the tympanic membrane (SPTM)	—	Appearance of otorrhea 4 to 30 days after the end of antibiotic treatment	—
Principi [22]	2016	Acute otitis media with spontaneous perforation of the tympanic membrane (AOM with SPTM)	—	—	Best antibiotic treatment
Sonsuwan [23]	2016	Acute otitis media with tympanic membrane perforation (AOMwiP)	—	—	—
Venekamp [24]	2016	Acute otitis media with discharge (AOMd)	—	—	Best antibiotic treatment
Yunfang [25]	2015	Acute otitis media with spontaneous otorrhea (AOMSO)	—	—	—
Rodrigues [26]	2013	Acute otitis media with spontaneous otorrhea (AOMSO)	—	—	—
Chen [27]	2013	Acute otitis media with spontaneous otorrhea (AOMSO)	—	—	—
Marchisio [28]	2013	Acute otitis media with spontaneous perforation of the tympanic membrane (AOM with SPTM)	≤ 3 days	—	—
Smith‐Vaughn [29]	2013	Acute otitis media with tympanic membrane perforation (AOMwiP)	≤ 6 weeks	—	Monitoring of antimicrobial profile and resistance
Grevers [30]	2012	Acute otitis media with tympanic membrane perforation (AOMwiP)	—	≥ 3 or more new episodes within the past 6 months, or ≥ 4 new episode within the past year	—
Marsh [31]	2012	Acute otitis media with tympanic membrane perforation (AOMwiP)	< 6 weeks	—	Monitoring of antimicrobial profile and resistance
Neumark [32]	2011	Acute otitis media with discharge (AOMd)	< 3 days	≥ 3 episodes within 6 months	—
Stamboulidis [33]	2011	Acute otitis media with discharge (AOMd)	—	—	—
Smith [4]	2010	Ear discharge	—	—	Best antibiotic treatment
Leibovitz [34]	2009	Acute otitis media with otorrhoea	≤ 7 days	—	—
Al‐Shawwa [35]	2005	Acute otitis media with otorrhoea	—	—	—
Berger [36]	1989	Acute otitis media with spontaneous perforation of the tympanic membrane (AOM with SPTM)	—	—	—

### 3.2 Consensus Exercise

During the first round of voting, agreement was established for the duration which should be used for acute ear discharge (≤ 6 weeks) and the definition of recurrent disease (4 or more episodes in 12 months) (Table 4). The terminology which should be used for acute ear discharge was found to be unanimous during the second round of voting (acute otitis media with discharge [AOMd]).

**TABLE 4:** Consensus voting by voting round (9‐point scale: 1 = extremely inappropriate, 5 = uncertain, and 9 = extremely appropriate).

Participant	EH	JN	MW	MK	JM	AM
Round 1 voting						
What terminology should we use to describe acute ear discharge secondary to a middle ear infection?						
ASOM	7	6	8	5	9	2
AOMd	9	9	5	8	1	9
What time frame differentiates acute from chronic discharge?						
≤ 2 weeks	7	6	6	3	1	3
≤ 6 weeks	8	9	8	8	1	8
≤ 12 weeks	3	4	2	4	9	1
How many episodes over what time period represents recurrent infections?						
Two or more episodes in 12 months	2	3	2	3	2	3
Three or more episodes in 12 months	7	6	2	3	5	3
Four or more episodes in 12 months	8	7	8	9	9	9
Round 2 voting						
What terminology should we use to describe acute ear discharge secondary to a middle ear infection?						
ASOM	5	7	6	5	9	2
AOMd	9	8	9	8	7	9

Abbreviations: AOMd, acute otitis media with discharge; ASOM, acute suppurative otitis media.

The opinion of the expert panel was that acute otitis media with discharge (AOMd) is ‘more relatable to patients and they might understand it a bit better’ compared to acute suppurative otitis media. The duration of less than or equal to 6 weeks was thought to ‘align with other definitions of chronic disease’ and would allow appropriate management in primary care prior to secondary care referral. The panel wanted to mirror the definition of recurrent AOM in that three or more episodes in 4 months or four or more episodes in 12 months indicates recurrent disease.

### 3.3 Research Priorities

Panellists selected research priorities from the list produced from the systematic review and their own experience prior to the consensus meeting. Identifying the best management option was selected by all participants (*n* = 6) followed by understanding the hearing impact (*n* = 4), determining when patients should be referred to secondary care (*n* = 4), monitoring antimicrobial resistance (*n* = 3), identifying causative factors for disease (*n* = 3), cost benefit of antibiotic treatment (*n* = 2), role of microbiology swabs (*n* = 2), and identifying antimicrobial pathogens (*n* = 1).

## 4 Discussion

Standardisation of disease terminology is essential for patient care, clinician communication, and research. Parents have identified that there is insufficient patient information material available for AOMd [3]. Understandably, it is difficult to create or search for a condition when there is no clear definition. The service user with lived experience on the expert panel was clear that any terminology should be easy to understand for patients and their families. Furthermore, the systematic review has identified the heterogeneity of terms used to describe acute paediatric ear discharge, which reinforces the need for standardisation.

Examining the trend of terms used within the literature, it appears that the use of AOMd is becoming more commonplace [3, 11, 15, 16]. This allows this subset of patients to be clearly differentiated from AOM without discharge. AOM is the most common cause of discharge in younger children; however, there are other causes which clinicians must consider. For example, cholesteatoma is an accumulation of squamous epithelium, which usually causes discharge resistant to treatment [37]. Older children can suffer from otitis externa causing otorrhoea, but the clinical presentation will likely differ from those with AOM. It can be challenging to determine the cause of the discharge, especially at the first presentation when the ear canal is full of pus. Patients are frequently treated empirically with oral or topical antibiotics [3].

Current NICE guidance states that a child with ear discharge for 2 weeks or more in duration should be classified as chronic suppurative otitis media (CSOM) and be referred to secondary care [38]. Our expert panel felt a diagnosis of ‘acute’ ear discharge should be used when otorrhoea persists for less than or equal to 6 weeks duration to allow for appropriate treatment in primary care before the child is referred to secondary care.

A patient and public engagement group composed of 6 parents of children with AOMd was consulted following the consensus exercise. All parents confirmed that the proposed terminology was understandable.

The limitations of this consensus exercise include the small size of the expert panel who all work within the NHS (UK). These views will not necessarily represent those from all countries. To diversify opinions, a multidisciplinary approach was taken to include as many stakeholders and expert specialists as possible. There were no deviations from the protocol for the RAND modification of the Nominal Group Technique [8, 9]. There is variation within the prior literature as to what is an appropriate group size. We took a pragmatic approach to panel size to ensure there were varied opinions, panellist availability, and consideration of session duration. In our experience, a panel of seven experts gave each member adequate time to share their opinions during a 2‐hour session.

This consensus exercise has outlined that acute ear discharge in children should be called acute otitis media with discharge (AOMd). Discharge can last for up to and including 6 weeks; thereafter it should be called CSOM. The definition of recurrent AOMd should follow three episodes within 6 months or four episodes within 12 months. Standardising terminology will help streamline patient care and improve homogeneity of future research. Future research priorities to be addressed, in this disease context, are understanding the best management strategy and the impact on hearing.

## Author Contributions

All authors meet the International Committee of Medical Journal Editors (ICMJE) guidance for authorship.

## Ethics Statement

Ethical approval was not required for this study as it did not directly collect data from patients.

## Conflicts of Interest

The authors declare no conflicts of interest.

## Peer Review

The peer review history for this article is available at https://www.webofscience.com/api/gateway/wos/peer‐review/10.1111/coa.70021.

## Data Availability

Data sharing not applicable to this article as no datasets were generated or analysed during the current study.
